# MTHFR 677TT is associated with decreased number of embryos and cumulative live birth rate in patients undergoing GnRHa short protocol: a retrospective study

**DOI:** 10.1186/s12884-022-04506-4

**Published:** 2022-03-01

**Authors:** Hong Zeng, Zefu Liu, Lei Zhang, Nenghui Liu

**Affiliations:** 1 Department of Reproductive Medicine Center, Xiangya Hospital, Central South University, Changsha, 410008 Hunan China; 2 Department of Reproductive Medicine Center, Foshan Maternal and Child Health Care Hospital, Southern Medical University, Foshan, 528000 Guangdong China; 3 Department of Reproductive Medicine Center, Nanfang Hospital, Southern Medical University, Guangzhou, 510515 Guangdong China; 4 Department of Urology, Sun Yat-sen University Cancer Center, Guangzhou, 510060 Guangdong China

**Keywords:** 5,10-methylenetetrahydrofolate reductase, Cumulative live birth rate, Retrospective analysis

## Abstract

**Background:**

Whether MTHFR C677T genotype affects pregnancy outcomes following assisted reproductive technology is conflicting. And the role of MTHFR C677T genotype on cumulative live birth has not been reported. This study aims to investigate the effect of MTHFR C677T genotype on cumulative live birth following in-vitro fertilization and embryo transfer (IVF-ET).

**Methods:**

This is a retrospective cohort study that includes 1173 women undergoing their first IVF-ET. We retrospectively compared the reproductive outcomes among the groups stratified by MTHFR C677T genotypes (677CC, 677CT, 677TT). We performed interaction analysis to detect the factor that interacts with the MTHFR C677T genotype. Poisson regression analyses were used to evaluate the associations between MTHFR C677T genotypes with the number of transferable embryos and the number of good-quality embryos. Cox regression analysis was used to evaluate the association between MTHFR C677T genotypes with cumulative live birth. All regression analyses were adjusted with the confounding factors which may independently impact reproductive outcomes.

**Results:**

There is a significant interactive effect of MTHFR 677TT genotype with GnRHa protocol on reproductive outcomes (P for interaction<0.05). MTHFR 677TT homozygous mutation was found to impact reproductive outcomes under GnRHa short protocol but not GnRHa long protocol. MTHFR 677TT is significantly associated with decreased number of transferable embryos (*p*-value=0.028), decreased number of good-quality embryos (*p*-value=0.005), and decreased cumulative live birth rate (*p*-value=0.024) in patients undergoing GnRHa short protocol. However, the clinical pregnancy rate, miscarriage rate and live birth rate at the first embryo transfer cycle were not significantly different between the groups under both protocols (*p*-values>0.05).

**Conclusions:**

MTHFR 677TT genotype is associated with decreased number of transferable embryos, decreased number of good-quality embryos, and decreased cumulative live birth rate in the first complete cycle in patients undergoing GnRHa short protocol.

**Supplementary Information:**

The online version contains supplementary material available at 10.1186/s12884-022-04506-4.

## Background

5,10-methylenetetrahydrofolate reductase (MTHFR) is a crucial enzyme for the metabolism of folic acid. MTHFR catalyzes 5,10-methylenetetrahydrofolate to 5-methylenetetrahydrofolate. The latter is the predominant circulating form of folate and is essential for the synthesis of nucleotide, re-methylation of homocysteine (hcy), methylation of DNA, proteins, neurotransmitters and phospholipids [1, 2]. Some genetic polymorphisms associated with deficiencies in the folic acid metabolic pathway are supposed to be a clinically genetic susceptibility factor for many diseases [3–7]. Among them, polymorphisms of MTHFR play essential roles. Though, more than 20 polymorphisms of MTHFR have been described [8]. MTHFR C677T (rs1801133) is one of the most investigated polymorphisms of MTHFR. The MTHFR 677C>T transition causes an alanine to valine replacement (Ala222Val) in the catalytic domain of MTHFR. MTHFR 677CT and MTHFR 677TT have in vitro MTHFR activity reduced by about 70% and 35%, respectively [9]. Reduced MTHFR activity is detrimental to the development of oocytes and embryos [10, 11]. Evidence showed that MTHFR 677C>T is associated with ovarian reserve, oocyte maturation, and embryo aneuploidy [12–14] and is linked to premature ovarian failure [15], recurrent miscarriages [16], and recurrent implantation failures [14, 17].

Accumulating evidence support that maternal variant in MTHFR C677T polymorphism is associated with poorer quality of oocytes and lower viability of embryos [13]. Paternal MTHFR C677T mutation is reported to be associated with the production of aneuploid embryos [13]. Because the quality of gamete or zygote is crucial for pregnancy outcomes. MTHFR C677T mutation is very likely to impact the IVF/ICSI-ET outcomes. Therefore, testing of MTHFR could be of great clinical value. However, most previous studies support that MTHFR C677T genotype is not associated with pregnancy outcomes such as clinical pregnancy rate, ongoing pregnancy rate, live birth rate following IVF/ICSI-ET [18–22]. A meta-analysis also reports that the clinical pregnancy rate following IVF-ET is not significantly associated with MTHFR C677T mutation [23]. As the quality of gamete or embryo is an independent factor for pregnancy outcomes following IVF/ICSI-ET. There is a contradiction that MTHFR C677T affects the quality of gamete or embryo but does not affect pregnancy outcomes. It is worth noting that most studies include patients with only one ET cycle or their first ET cycle. Commonly, we will transfer the best embryos in the first ET cycle as we get multiple oocytes or embryos following one ovarian stimulation, and this is one of the potential reasons that the effect of MTHFR C677T genotype on pregnancy outcomes is not observed in those studies. Besides, high heterogeneity exists among the previous studies, including different ethnic backgrounds and populations, different inclusion and exclusion criteria. Most of the studies included small sample size and hardly control the confounding factors. However, whether the MTHFR C677T genotype impacts the quality of embryos and the cumulative pregnancy outcomes over the subsequent ET cycles has not been reported. Since ovarian stimulation can produce multiple oocytes and embryos at a time, it is difficult to reflect the cumulative effect of a genetic factor on embryos if only the pregnancy outcome after the first embryo transfer is concerned, rather than the cumulative live birth rate after an ovulation cycle. Cumulative live birth rate reflects the overall outcome of a treatment cycle and is, therefore, the most important indicator of clinical treatment success rate. Therefore, we perform this retrospective analysis to evaluate the effect of MTHFR C677T polymorphism on the long-term outcome as cumulative live birth.

## Methods

### Study design

This is a retrospective cohort study that includes 1173 women undergoing their first fresh autologous IVF/ICSI cycles at the Reproductive Medicine Center of Xiangya Hospital from 2015 to 2017. The study was approved by the ethical committee of Xiangya Hospital. Data were retrospectively collected through the electronic database. The patients were followed up for a minimum of 2 years and a maximum of 5 years. The observational endpoint is live birth or uses up all the embryos in the first ovarian stimulation cycle without a live birth. We compared the ART outcomes between the groups stratified by MTHFR C677T genotypes (677CC, 677CT, 677TT). We performed interaction analysis to detect the factor that interacts with the MTHFR C677T genotype. We performed subgroup analysis based on different protocols. Poisson regression analyses were used to evaluate the associations between MTHFR C677T genotypes with the number of transferable embryos and the number of good-quality embryos. Cox regression analysis was used to evaluate the association between MTHFR C677T genotypes with cumulative live birth. All the regression analyses were adjusted with confounding factors including age, BMI, infertility cause, stimulation protocol, and infertility type.

### Inclusion and exclusion criteria

The inclusion criteria are: patients undergoing their first fresh autologous IVF/ICSI-ET cycles; the couples are Chinese Han nationality; women were transferred with 1~2 morphologically good-quality embryos at Day 3 in the first ET cycle; the infertility cause is the male factor or simple female tubal factor; the stimulation protocol is standard GnRHa long protocol or GnRHa short protocol. Patients were excluded if age > 45 years, BMI < 18 kg/m^2^ or > 25 kg/m^2^. Patients with abnormal karyotype, thyroid dysfunction, endometriosis, severe hydrosalpinx, intrauterine adhesions, uterine myoma>5cm, or donor cycles were also excluded. Measurement of MTHFR C677T genotype is routinely recommended for every patient planning IVF/ICSI-ET in our center in recent six years. Except for very few patients who refused the test, most of the patients who planned to receive IVF/ICSI-ET underwent the test of MTHFR C677T genotype, especially those with a history of adverse pregnancy such as spontaneous abortion. We excluded patients in the initial cohort if principal variables such as MTHFR C677T genotype are missing.

### ART procedure

Controlled ovarian stimulation (COS) was performed using the recombinant FSH (rFSH) or urinary FSH (uFSH) with or without human Menopausal Gonadotrophin (HMG) under pituitary suppression by Gonadotrophin releasing hormone analogue (GnRHa). The dose of starting gonadotrophin (Gn) was decided based on maternal age, antral follicle count (AFC), basal FSH concentration, AMH concentration, and BMI. One hundred fifty to three hundred IU recombinant FSH or human menopausal gonadotrophin were used to promote follicle development. The follicular development was monitored and the Gn dose was adjusted according to patients’ response during COS. Follicular development measurement was performed every 2~3 days by transvaginal ultrasound and serum estradiol (E_2_). Follicle was triggered with 5000~10,000 IU of hCG. Oocytes were picked up 36 h after hCG trigger. Luteal phase support was conventionally performed on the day of OPU. Embryos were transferred on Day 3. All the patients were routinely supplemented with 800 μg folic acid daily when starting ovarian stimulation and last for three months if pregnancy is achieved. MTHFR C677T genotype does not affect any clinical management during ART procedure.

### Outcomes and definitions

The primary outcome is the cumulative live birth rate at the first complete cycle. The secondary outcomes are the number of transferable embryos and the number of good-quality embryos. The number of oocytes and clinical pregnancy outcomes (include hCG positive rate, clinical pregnancy rate, miscarriage rate, live birth rate) at the first ET cycle are also reported.

We defined the hCG positive cases as increased serum hCG levels >10 IU/L at 10-13 days after embryo transfer. Ultrasonographic visualization of gestational sacs at 4~5 weeks after embryo transfer confirmed clinical pregnancy. Miscarriage was defined as a spontaneous clinical pregnancy loss before 24 gestational weeks (GW). We defined the live birth as the birth of at least one newborn after 24 GW that exhibits any sign of life. The cumulative live birth was defined as the live birth following a first fresh IVF cycle plus all additional frozen cycles in which the transferred embryos were from the first ovarian stimulation.

### Statistical analysis

The categorical variables were presented as percentages and frequencies. The continuous variables were presented as means and standard deviations (SDs) or interquartile ranges (IQRs) depending on the data distribution. We compared categorical variables between groups using the χ^2^ test. We compared continuous variables using the analysis of variance (ANOVA) or the Kruskal-Wallis test according to distribution type. Post hoc multiple comparisons were performed if the crude *p*-value is statistically significant (*p*-value<0.05). We use the Poisson regression analysis to analyze the association of MTHFR C677T genotype with the number of transferable embryos and the number of good-quality embryos. We use the Cox regression analysis to analyze the association of MTHFR C677T genotype with CLB at the first complete cycle. All multivariate regression models were adjusted by the confounding factors that may independently affect ART outcomes. Variables included in the multivariate model are maternal age, BMI, Infertility cause, infertility type, and stimulation protocol. We also conducted the interaction analysis stratified by maternal age (<35 years, ≥35 years), BMI (18.5-20 kg/m^2^, 20-23 kg/m^2^, 23-25 kg/m^2^), stimulation protocol (GnRHa long protocol, GnRHa short protocol), infertility cause (male factor and female tubal factor), and infertility type (primary infertility and secondary infertility). Each stratification was adjusted for all factors in the multivariate model except for the stratification factor itself. Hardy-Weinberg equilibrium (HWE) was used to detect the distribution of MTHFR C677T genotypes with the Log-likelihood ratio test. Deviation from HWE was confirmed if *p*-value < 0.05. The statistical power was estimated by the "pwr" R package. For ANOVA analyses, we used the "pwr.anova.test" function to calculate the power. For chi-squared tests, we used the "pwr.chisq.test" function to calculate the power. For all calculations, the significance level was set as 0.05. All statistical analyses were 2-sided, and we considered a *p*-value of less than 0.05 to be statistically significant. The statistical analyses were performed by R (version 4.0.5, released on 2021-03-31, www.R-project.org).

## Results:

**Fig. 1:**
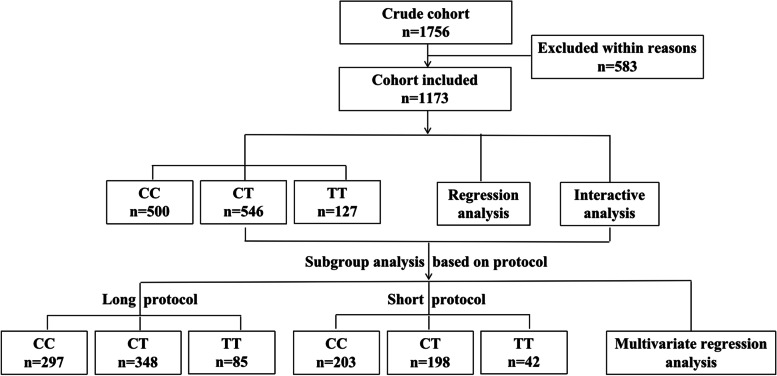
Flow chart of the study

After exclude patients who were not first ART cycles, 1756 women were included in the initial cohort. The following patients were further excluded: cycle cancelled (*n*=23), all embryos cryopreserved (*n*=79), abnormal karyotype (*n*=19), not Chinese Han population (*n*=34), thyroid dysfunction (*n*=75), severe hydrosalpinx (*n*=21), intrauterine adhesion (*n*=47), uterine myoma>5cm (*n*=26), BMI<18kg/m^2^ or BMI >25 kg/m^2^ (*n*=51), maternal age>45 years (*n*=38), embryos not transferred at D3 (*n*=43), loss to follow-up (*n*=12), incomplete data (*n*=23), endometriosis (*n*=63), other stimulation protocols (*n*=27), induced labor due to congenital malformations (*n*=2). After all exclusions, a total of 1173 women remained. Based on the results of MTHFR C677T genotyping, 500 women were MTHFR 677CC, 546 women were MTHFR 677CT, 127 women were MTHFR 677TT. There was no deviation from HWE in the included population (*p*-value = 0.22). The flowchart of the study is shown in Fig. 1.

### Baseline characteristics

Baseline parameters including maternal age, BMI, duration of infertility, infertility type, infertility cause, stimulation protocol, basal FSH level, basal LH level, Gn starting dose, Gn total dose, days of stimulation, endometrial thickness at the first fresh cycle, endometrial pattern at the first fresh cycle, fertilization method, and the number of transferred embryos. All the baseline parameters were not significantly different among the three groups except for the infertility cause (Table 1). As the following interactive analysis indicate that the MTHFR C677T genotype is interactive with ovarian stimulation protocol on ART outcomes. We further performed subgroup analysis based on stimulation protocol. For GnRHa long protocol, the baseline parameters were not significantly different between the three groups except for the infertility cause and fertilization method (Table 1). For GnRHa short protocol, all the baseline parameters were not significantly different among the three groups (Table 1).

**Table 1:** Baseline characteristics stratified by MTHFR C677T genotype

	Total *N*=1173				Long protocol *N*=730				Short protocol *N*=443			
	CC *N*=500	CT *N*=546	TT *N*=127	*P*-value	CC *N*=297	CT *N*=348	TT *N*=85	*P*-value	CC *N*=203	CT *N*=198	TT *N*=42	*P*-value
Age (Year)	30.52 (4.90)	30.65 (4.89)	30.66(4.69)	0.892	29.57 (4.09)	29.80 (4.23)	30.02 (4.69)	0.623	31.91 (5.62)	32.15 (5.58)	31.95 (4.48)	0.906
BMI (kg/m^2^)	21.47 (1.82)	21.39 (1.90)	21.50 (1.86)	0.732	21.43 (1.89)	21.30 (1.89)	21.34 (1.90)	0.666	21.52 (1.72)	21.55 (1.92)	21.82 (1.73)	0.622
Infertility Type:				0.695				0.936				0.497
Primary infertility	231 (46.20%)	239 (43.77%)	59 (46.46%)		144 (48.48%)	164 (47.13%)	40 (47.06%)		87 (42.86%)	75 (37.88%)	19 (45.24%)	
Secondary infertility	269 (53.80%)	307 (56.23%)	68 (53.54%)		153 (51.52%)	184 (52.87%)	45 (52.94%)		116 (57.14%)	123 (62.12%)	23 (54.76%)	
Infertility Year	4.28 (3.49)	4.18 (3.15)	4.31 (3.36)	0.842	3.92 (3.10)	4.19 (3.10)	3.98 (2.49)	0.513	4.81 (3.95)	4.15 (3.25)	5.00 (4.62)	0.145
Infertility cause:				0.021				0.035				0.282
Male factor	61 (12.20%)	39 (7.14%)	13 (10.24%)		38 (12.79%)	24 (6.90%)	7 (8.24%)		23 (11.33%)	15 (7.58%)	6 (14.29%)	
Female factor	439 (87.80%)	507 (92.86%)	114 (89.76%)		259 (87.21%)	324 (93.10%)	78 (91.76%)		180 (88.67%)	183 (92.42%)	36 (85.71%)	
Protocol:				0.181								
Long protocol	297 (59.40%)	348 (63.74%)	85 (66.93%)									
Short protocol	203 (40.60%)	198 (36.36%)	42 (33.07%)									
Endometrial Thickness	10.53 (2.20)	10.67 (2.11)	10.64 (2.25)	0.596	10.92 (2.16)	11.07 (2.21)	10.96 (2.37)	0.675	9.97 (2.14)	9.96 (1.71)	10.00 (1.86)	0.994
Endometrial Pattern:				0.494				0.912				0.257
A	224 (44.80%)	239 (43.77%)	64 (50.39%)		144 (48.48%)	165 (47.41%)	42 (49.41%)		80 (39.41%)	74 (37.37%)	22 (52.38%)	
B	256 (51.20%)	277 (50.73%)	59 (46.46%)		143 (48.15%)	166 (47.70%)	39 (45.88%)		113 (55.67%)	111 (56.06%)	20 (47.62%)	
C	20 (4.00%)	30 (5.49%)	4 (3.15%)		10 (3.37%)	17 (4.89%)	4 (4.71%)		10 (4.93%)	13 (6.57%)	0 (0.00%)	
basalFSH	6.59 (1.56)	6.62 (1.70)	6.54 (1.50)	0.841	6.46 (1.47)	6.57 (1.75)	6.17 (1.30)	0.115	6.77 (1.67)	6.73 (1.60)	7.29 (1.62)	0.122
basalLH	5.85 (3.14)	5.52 (3.19)	5.59 (3.04)	0.229	5.93 (2.95)	5.63 (3.37)	5.60 (2.91)	0.446	5.73 (3.41)	5.32 (2.85)	5.57 (3.32)	0.421
Gn start dose	164 (50.44)	165 (51.22)	162 (52.19)	0.736	157 (47.24)	160 (48.05)	155 (49.87)	0.516	175 (53.17)	174 (55.37)	175 (54.73)	0.995
Gn Day	10.5 (2.47)	10.5 (2.38)	10.4 (2.18)	0.947	11.13 (2.37)	11.06 (2.13)	10.99 (2.01)	0.842	9.57 (2.33)	9.41 (2.42)	9.29 (2.09)	0.701
Gn total dose	1917 (662)	1925 (662)	1845 (626)	0.455	1980 (639)	2009 (668)	1894 (647)	0.345	1826 (685)	1779 (627)	1746 (574)	0.669
Fertilization method				0.149				0.024				0.655
IVF	342(68.40%)	403(73.81%)	88(69.29%)		200(67.34%)	258(74.14%)	60(70.59%)		142(69.95%)	145(73.23%)	28(66.67%)	
ICSI	153(30.60%)	132(24.18%)	37(29.13%)		96(32.32%)	81(23.28)	23(27.06)		57(28.08%)	51(25.76%)	14(33.33%)	
IVF+ICSI	5(1.00%)	11(2.01%)	2(1.57%)		1(0.34%)	9(2.59%)	2(2.35%)		4(1.97%)	2(1.01%)	0(0.00%)	
No of ET				0.798				0.293				0.276
1	44 (8.80%)	44 (8.06%)	9 (7.09%)		17 (5.72%)	27 (7.76%)	3 (3.53%)		27 (13.30%)	17 (8.59%)	6 (14.29%)	
2	456 (91.20%)	502 (91.94%)	118 (92.91%)		280 (94.28%)	321 (92.24%)	82 (96.47%)		176 (86.70%)	181 (91.41%)	36 (85.71%)	

*Note*: The continuous variables were presented as mean (SD) and analyzed by ANOVA analysis, the categorical variables were presented as percentages (frequencies) and analyzed by chi-square test. *P* < 0.05 was considered as statistically significant. *MTHFR* 5,10-Methylenetetrahydrofolate reductase, *BMI* Body mass index, *SD* Standard deviation, *FSH* Follicle stimulating hormone, *LH* Luteinizing hormone, *IVF* In-vitro fertilization, *ICSI* Intracytoplasmic sperm injection, *Gn* Gonadotrophin, *No* Number

### Laboratory outcomes

The laboratory outcomes include the number of oocytes obtained, the number of transferable embryos, and the number of good-quality embryos. All the laboratory outcomes are not significantly different between the CC, CT, and TT groups (Table 2). However, when patients are stratified by stimulation protocol, results showed that the number of transferable embryos was significantly different between the three group under the GnRHa short protocol (*p*-value=0.028). Post hoc multiple comparisons showed that the number of transferable embryos was significantly lower in the TT group compared to the CT group (*p*-value=0.001) or CC group (*p*-value =0.001) under the GnRHa short protocol, while the number of transferable embryos was not significantly different between the CT group and CC group (*p*-value=0.858). The number of good-quality embryos was significantly different between the three groups under the GnRHa short protocol (*p*-value=0.005). Post hoc multiple comparisons showed that the number of good-quality embryos was lower in the TT group compared to the CT group (*p*-value<0.001) or CC group (*p*-value<0.001) in women undergoing GnRHa short protocol, while the number of good-quality embryos was not significantly different between the CT group and CC group (*p*-value =0.941) (Table2). The laboratory outcomes were not significantly different between the groups in women undergoing GnRHa long protocol (Table2). As the sample size in the TT group under the GnRHa short protocol is relatively small, the statistical power was calculated for each outcome include the number of transferrable embryos and the number of good-quality embryos under the GnRHa short protocol. As the statistical power estimation curve (Supplementary figure 1) shows, the power for the number of transferrable embryos is 0.67, 0.78, 0.84, 0.87 when the significance level is 0.05, 0.10, 0.15, 0.20, respectively; the statistical power for the number of good-quality embryos is 0.98, 0.99, 1.00, 1.00 when the significance level is 0.05, 0.10, 0.15, 0.20, respectively. From the above results, we can tell that, when the false positive rate is less than 5%, there is a 68% chance that the MTHFR C677T genotype is truly associated with the number of transferrable embryos under the GnRHa protocol; there is a 98% chance that MTHFR C677T genotype is truly associated with the number of good-quality embryos under the GnRHa short protocol. When the false positive rate is less than 10%, there is a 78% chance that the MTHFR C677T genotype is truly associated with the number of transferrable embryos under the GnRHa protocol; there is a 99% chance that MTHFR C677T genotype is truly associated with the number of good-quality embryos under the GnRHa short protocol.

**Table 2:** Laboratory outcomes stratified by MTHFR C677T genotype

	Total *N*=1173				Long protocol *N*=730				Short protocol *N*=443			
	CC *N*=500	CT *N*=546	TT *N*=127	*P*-value	CC *N*=297	CT *N*=348	TT *N*=85	*P*-value	CC *N*=203	CT *N*=198	TT *N*=42	*P*-value
Oocytes	10.33 (4.41)	10.14 (4.24)	9.83 (4.00)	0.463	10.64 (4.39)	10.53 (4.22)	10.33 (4.08)	0.838	9.89 (4.41)	9.44 (4.20)	8.81 (3.65)	0.269
Transferable embryos	4.28 (2.57)	4.17 (2.41)	3.88 (2.21)	0.254	4.23 (2.54)	4.09 (2.31)	4.20 (2.38)	0.763	4.36 (2.62) a^NS^	4.31 (2.58) b^**^	3.24 (1.66) c^**^	0.028
Good-quality embryos	3.88 (2.35)	3.80 (2.23)	3.40 (1.92)	0.102	3.85 (2.30)	3.74 (2.14)	3.76 (2.06)	0.796	3.92 (2.42) a^NS^	3.90 (2.40) b^***^	2.67 (1.34) c^***^	0.005

*Note*: The continuous variables were presented as mean (SD) and analyzed by ANOVA analysis. *P* < 0.05 was considered as statistically significant. *MTHFR* 5,10-Methylenetetrahydrofolate reductase. a: CC versus CT. b: CT versus TT. c: TT versus CC. * *P* < 0.05, ** *P* < 0.01, *** *P* < 0.001, NS: not significant. *P*-values of post-hoc multivariate comparisons under GnRHa short protocol: For the number of transferable embryos: CC versus CT (*p*-value=0.858), CT versus TT (*p*-value=0.001); TT versus CC (*p*-value=0.001). For the number of good-quality embryos: CC versus CT (*p*-value=0.943), CT versus TT (*p*-value<0.001), TT versus CC (*p*-value<0.001)

### Clinical pregnancy outcomes

The clinical pregnancy outcomes include the hCG positive rate, clinical pregnancy rate, miscarriage rate, live birth rate at the first ET cycle, and the cumulative live birth rate at the first complete cycle. All the clinical outcomes were not significantly different between the CC, CT, and TT groups (Table 3). However, when patients are stratified by stimulation protocol, results showed that the CLBR was significantly different between the three groups (*p*-value=0.024) under GnRHa short protocol. Post hoc multiple comparisons showed that the CLBR was significantly lower in the TT group compared to the CC group (*p*-value=0.035), CLBR was significantly lower in the CT group compared to the CC group (*p*-value =0.048), CLBR was not significantly different between the CT group and TT group (*p*-value =0.364) (Table 3). While the clinical outcomes were not significantly different between the groups in women undergoing GnRHa long protocol (Table 3). As the sample size in the TT group under the GnRHa short protocol is relatively small, the statistical power was calculated for CLBR under the GnRHa short protocol. As the statistical power estimation curve (Supplementary figure 1) shows, the statistical power for cumulative live birth rate is 0.69, 0.79, 0.84, 0.88 when the significance level is 0.05, 0.10, 0.15, 0.20, respectively. From the above results, we can tell that, when the false positive rate is less than 5%, there is a 69% chance that the MTHFR C677T genotype is truly associated with CLBR. when the false positive rate is less than 10%, there is a 79% chance that the MTHFR C677T genotype is truly associated with CLBR.

**Table 3:** Clinical pregnancy outcomes stratified by MTHFR C677T genotype.

	Total *N*=1173				Long protocol *N*=730				Short protocol *N*=443			
	CC *N*=500	CT *N*=546	TT *N*=127	*P*-value	CC *N*=297	CT *N*=348	TT *N*=85	*P*-value	CC *N*=203	CT *N*=198	TT *N*=42	*P*-value
hCG positive rate	301 (60.20%)	325 (59.52%)	73 (57.48%)	0.855	200 (67.34%)	222 (63.79%)	55 (64.71%)	0.635	101 (49.75%)	103 (52.02%)	18 (42.86%)	0.553
CPR	256 (51.20%)	280 (51.28%)	63 (49.61%)	0.941	177 (59.60%)	200 (57.47%)	49 (57.65%)	0.853	79 (38.92%)	80 (40.40%)	14 (33.33%)	0.694
Miscarriage rate	31 (6.20%)	27 (4.95%)	8 (6.30%)	0.639	18 (6.06%)	19 (5.46%)	6 (7.06%)	0.843	13 (6.40%)	8 (4.04%)	2 (4.76%)	0.561
LBR	225 (45.00%)	245 (44.87%)	52 (40.94%)	0.694	155 (52.19%)	178 (51.15%)	40 (47.06%)	0.706	70 (34.48%)	67 (33.84%)	12 (28.57%)	0.759
CLBR	287 (57.40%)	287 (52.56%)	67 (52.76%)	0.263	185 (62.29%)	208 (59.77%)	54 (63.53%)	0.725	102 (50.25%)a^*^	79 (39.90%)b ^NS^	13 (30.95%)c^*^	0.024

*Note*: The categorical variables were presented as percentages (frequencies) and analyzed by chi-square test. *P* < 0.05 was considered as statistically significant. *MTHFR* 5,10-Methylenetetrahydrofolate reductase, *hCG* Human chorionic gonadotrophin, *CPR* Clinical pregnancy rate, *LBR* Live birth rate, *CLBR* Cumulative live birth rate. a: CC versus CT (*p*-value=0.048). b: CT versus TT (*p*-value=0.364). c: TT versus CC (*p*-value=0.035). * *P* < 0.05, NS: not significant.

### Interactive analysis

For the number of transferable embryos, we found that the MTHFR C677T genotype was significantly interactive with the stimulation protocol (P for interaction: 0.020). Results for multivariate regression analysis on the number of transferable embryos considering the interactive effect or not were shown in Supplementary Table S1. For the number of good-quality embryos, results showed that the MTHFR C677T genotype was significantly interactive with stimulation protocol (P for interaction: 0.005). Results for multivariate regression analysis on the number of good-quality embryos considering the interactive effect or not were shown in Supplementary Table S2. For cumulative live birth, we also found that the MTHFR C677T genotype was significantly interactive with the stimulation protocol (P for interaction: 0.013). Results for multivariate regression analysis on CLB considering the interactive effect or not were shown in Supplementary Table S3. Taken together, results of the interactive analysis indicate that MTHFR C677T exert different effects under different ovarian stimulation protocols. Therefore, we further performed the multivariate regression analyses stratified by different ovarian stimulation protocols.

### Multivariate regression analysis

**Fig. 2:**
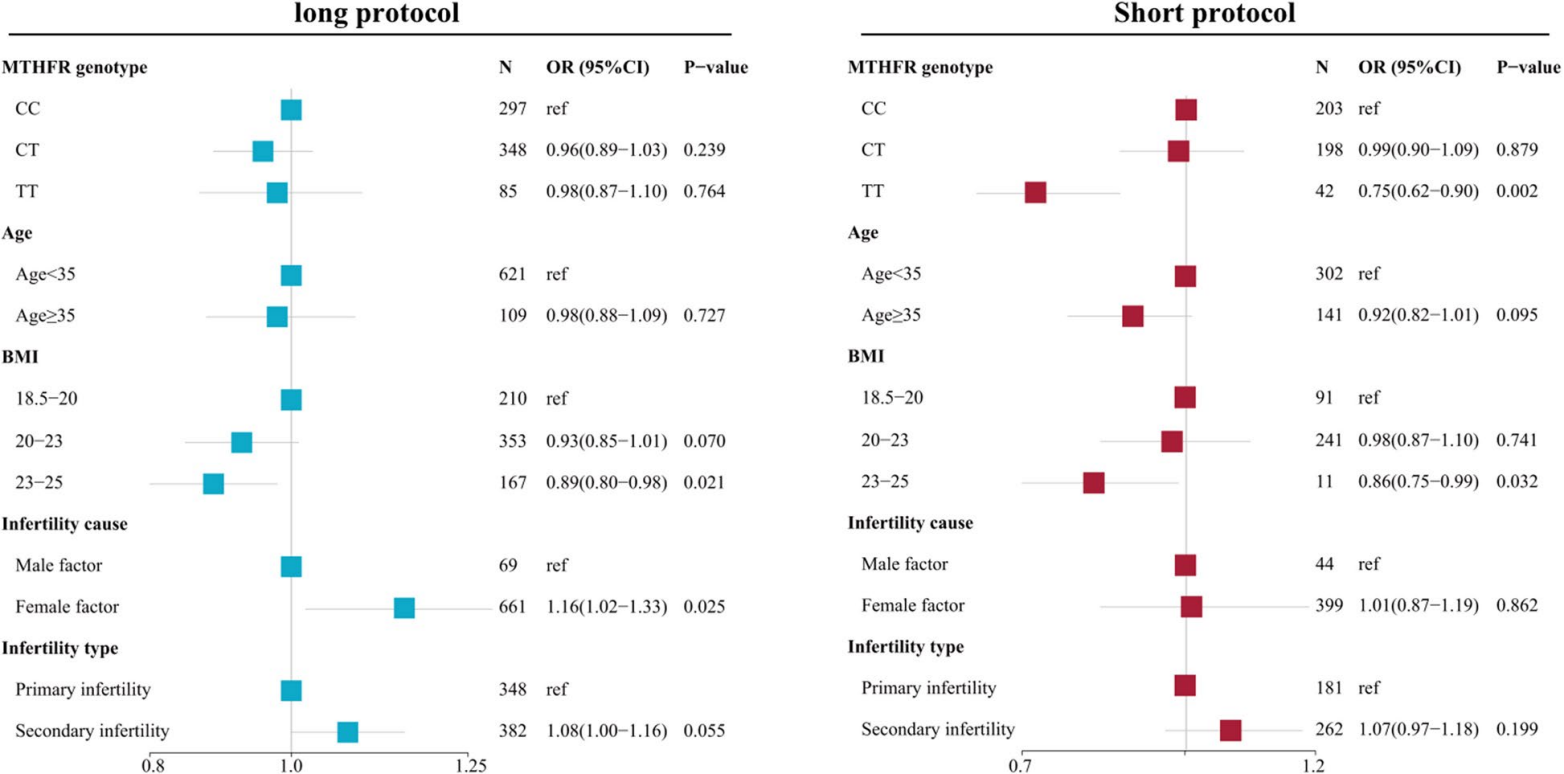
Forest plot showing the association of MTHFR C677T genotype with the number of transferable embryos by Poisson regression analysis adjusted by Age, BMI, infertility cause, and infertility type under GnRHa long protocol and short protocol

**Fig. 3:**
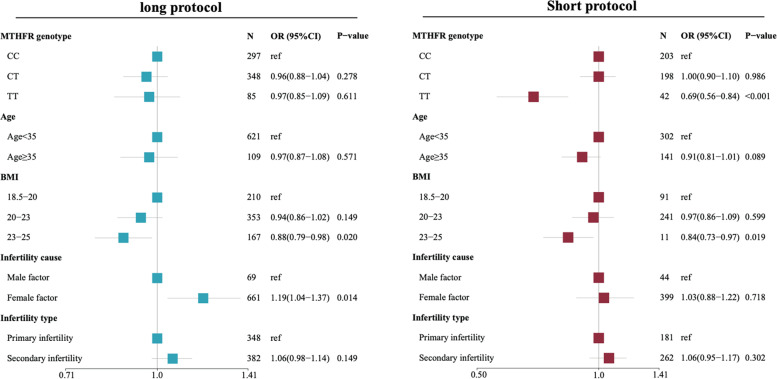
Forest plot showing the association of MTHFR C677T genotype with the number of good-quality embryos by Poisson regression analysis adjusted by Age, BMI, infertility cause, and infertility type under GnRHa long protocol and short protocol

**Fig. 4:**
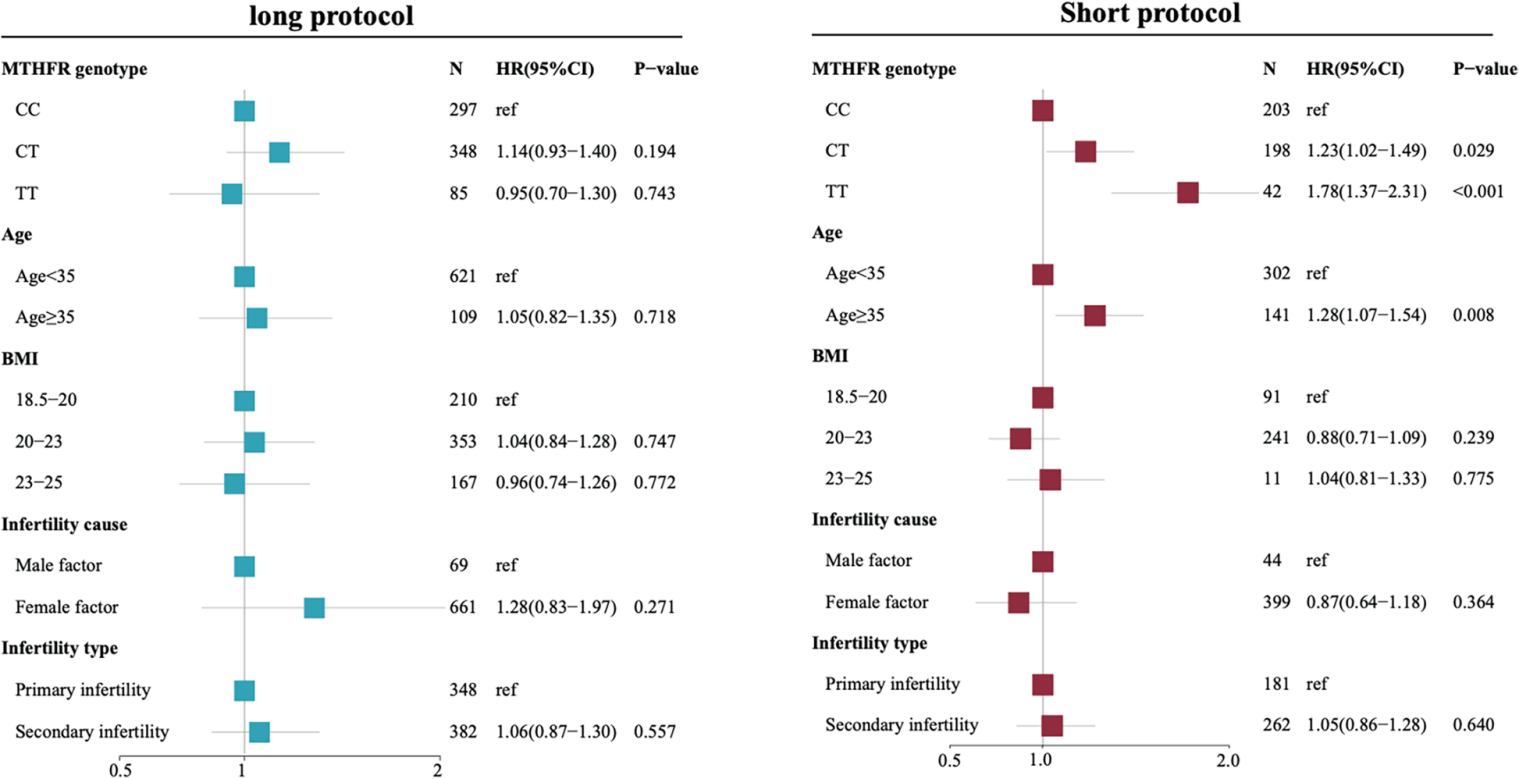
Forest plot showing the association of MTHFR C677T genotype with the cumulative live birth by Cox regression analysis adjusted by Age, BMI, infertility cause, and infertility type under GnRHa long protocol and short protocol

We performed the multivariate regression analysis to control the effect of confounding factors and explore the associations between the MTHFR C677T  genotype with the number of transferable embryos and the number of good-quality embryos, and cumulative live birth at the first complete cycle. For GnRHa long protocol, results showed that MTHFR C677T genotype was not significantly associated with the number of transferable embryos (TT: OR 0.98, 95%CI 0.87-1.10, *p*-value=0.764; CT: OR 0.96, 95%CI 0.89-1.03, *p*-value=0.239), the number of good-quality embryos (TT: OR 0.97, 95%CI 0.85-1.09, *p*-value=0.611; CT: OR 0.96, 95%CI 0.88-1.04, *p*-value=0.278), and cumulative live birth (TT: HR 0.95, 95%CI 0.70-1.30, *p*-value=0.743; CT: HR 1.14, 95%CI 0.94-1.40, *p*-value=0.194). For GnRHa short protocol, results showed that MTHFR 677TT homozygote was significantly associated with the lower number of transferable embryos (OR 0.75, 95%CI 0.62-0.90, *p*-value=0.002), lower number of good-quality embryos (OR 0.69, 95%CI 0.56-0.84, *p*-value<0.0001), and more ET cycles are needed to reach cumulative live birth (TT: HR 1.78, 95%CI 1.37-2.31, *p*-value<0.001). Results of multivariate Poisson regression analysis reveals the association of MTHFR C677T genotype with the number of transferrable embryos were shown in Fig. 2. Results of multivariate Poisson regression analysis reveals the association of MTHFR C677T genotype with the number of good-quality embryos were shown in Figure 3. Results of multivariate COX regression analysis reveals the association of MTHFR C677T genotype with cumulative live birth were shown in Fig. 4.

## Discussion

MTHFR is a pivotal gene for folate metabolism and is essential for human reproduction. MTHFR genetic mutation is linked to various reproduction-related diseases. MTHFR C677T is one of the most investigated polymorphisms of MTHFR. Therefore, testing of MTHFR C677T polymorphism could be of great clinical value. However, the role of MTHFR C677T polymorphism on outcomes following IVF/ICSI was indefinite. Zeng et.al reported that MTHFR C677T polymorphism is associated with a poor prognosis of COH oocytes. Pavlik et.al reported that MTHFR 677TT homozygous mutation is associated with a significantly lower number of oocytes than was expected based on the AMH concentrations [24]. Evidence also supports that maternal variant in MTHFR C677T polymorphism is associated with poorer quality of oocytes and lower viability of embryos [13]. Moreover, accumulating studies prove that paternal MTHFR 677TT homozygous mutation is significantly associated with male infertility [12, 25]. Parent MTHFR genotypes were shown to affect the production of aneuploid embryos [13]. While supplementation of folic acid for 3 months could improve the seminal parameters and decrease seminal MDA and sperm DNA fragmentation index in patients with MTHFR 677TT genotype as well as tend to decrease the spontaneous pregnancy rate and increase the live birth rate [26]. Though the current evidence indicates that the MTHFR 677TT genotype affects the ovarian response during ovarian stimulation and quality of gametes/embryos, however, the effect of the MTHFR 677TT genotype on pregnancy outcomes following IVF/ICSI-ET was not supported by the previous studies. A total of six studies investigated the role of maternal MTHFR C677T polymorphism on pregnancy outcomes following IVF/ICSI-ET, five of them reported no significant associations between maternal MTHFR 677TT genotype with pregnancy outcomes concerning clinical pregnancy rate, ongoing pregnancy rate, or live birth rate.

Our study found that the MTHFR 677TT genotype significantly decreased the number of transferable embryos, the number of good-quality embryos, and CLBR at the first complete cycle in patients undergoing GnRHa short protocol. While the clinical pregnancy rate, miscarriage rate and LBR at the first ET cycle were not significantly different between the groups. Our regression analyses adjusted by confounding factors also confirm the association of MTHFR 677TT homozygous mutation with a lower number of transferable embryos, lower number of good-quality embryos, and more ET cycles needed to achieve a live birth. Different from the previous studies, we found that the ovarian stimulation protocol is significantly interactive with the MTHFR C677T genotype on pregnancy outcomes as our results showed that, the MTHFR 677TT genotype is significantly associated with decreased number of transferable/good-quality embryos and cumulative live birth rate in patients undergoing GnRHa short protocol. However, MTHFR 677TT is not significantly associated with ART outcomes in patients undergoing GnRHa long protocol.

Gonadotrophin-releasing hormone agonists (GnRHa) are commonly used in assisted reproduction technology (ART) cycles to prevent a luteinizing hormone (LH) surge during controlled ovarian hyperstimulation (COH) before planned oocyte retrieval. For long protocol, GnRHa is administered at least two weeks before starting stimulation and continued up until HCG is given. For short protocol, GnRHa is administered from day one or two of the menstrual cycle and continued with stimulation until the day of HCG. GnRHa long protocol has longer and deeper pituitary downregulation compared to GnRHa short protocol. Longer pituitary downregulation significantly increased the number of mature oocytes [27], implantation rate, the number of good-quality embryos [28], clinical pregnancy rate, and live birth rate [29]. Recent studies support that longer pituitary downregulation favors ART outcomes regardless of age or ovarian response. Ou et al. found that the number of oocytes retrieved, MII oocytes, high-quality embryos, implantation rate, and clinical pregnancy rate in the long protocol group were all significantly greater than those in the short protocol group for all age ranges [30]. A Cochrane meta-analysis reported that GnRHa long protocol significantly increases the clinical pregnancy rate compared to GnRHa short protocol with moderate-quality evidence [31]. Several reasons help to explain why the long protocol was better: (1) promote better follicular synchronization, in turn leading to an increased number of oocytes; (2) improve the quality of oocytes and embryos; (3) improve the receptivity of the endometrium. Our results showed that the MTHFR 677TT genotype might have an additive effect with the GnRHa short protocol on the number and quality of embryos and cumulative live birth. MTHFR 677TT genotype is reported to be associated with ovarian reserve, oocyte maturation, and embryo aneuploidy [10–14]. Our results suggest that patients with MTHFR 677TT genotype is more vulnerable to GnRHa short protocol, thus leading to decreased number of transferable embryos and poorer embryo quality, and the CLBR is decreased, therefore. Maybe the influence of MTHFR genetic variations on cellular metabolic kinetics and biology was different under different protocols. However, the underlying molecular reason is still not clear.

It is worth noting that the clinical outcomes at the first ET cycle were not significantly different between the groups under both protocols. We think it is largely because that the patients included in the study are all transferred with 1 or 2 good-quality embryos at the first ET cycle. The reason we exclude the patients who do not transfer with a good embryo is to control the influence of embryo factors on reproductive outcomes of the first ET cycle. Thus, the clinical difference could not be detected due to the control of embryo factor as well as the baseline characteristics including age, BMI, stimulation protocol infertility cause, and infertility type. The clinical outcomes at the first ET cycle of our study are similar to most of the previous studies. However, in the subsequent transfers without controlling the embryo factors, we observed a significantly decreased cumulative live birth rate in patients with homozygous mutation under GnRHa short protocol. Although this difference was only observed in the GnRHa short protocol. Combined with the results that homozygous mutation of MTHFR 677TT, significantly reduced the number of good-quality embryos, we speculated that MTHFR 677TT mutation may affect pregnancy outcomes mainly by decreasing embryo quality. Besides, increasing evidence indicates that the T allele of the C677T MTHFR polymorphism is associated with hyperhomocysteinemia [32–35], vitamin D concentrations and natural killer cell cytotoxicity [16], all of these factors could affect reproductive outcomes.

As far as we know, this is the first study that investigates the effect of MTHFR C677T polymorphism on the cumulative live birth rate. Summarizing the previous researches and our study, we infer that MTHFR C677T polymorphism is more likely to impact reproduction in a homozygous model. As most of the studies indicate a deleterious effect of MTHFR 677TT homozygous mutation compared to MTHFR 677CC wild genotype. While few studies reported the deleterious effect of MTHFR 677CT heterozygous mutation compared to MTHFR 677CC wild genotype.

One value of this study is that we found the interactive effect of the MTHFR genotype with the GnRHa protocol. MTHFR 677TT genotype significantly decreased the number of transferable embryos, the number of good-quality embryos, and cumulative live birth under the GnRHa short protocol but not GnRHa long protocol. Therefore, we should be very cautious when applying GnRHa short protocol for women with MTHFR 677TT genotype. However, whether MTHFR C677T genotype affects reproductive outcomes under antagonist protocol or other protocols needs to be investigated. Another advantage of this study is that we used a rigorous statistical method to control the influences of the confounding factors. Besides, the sample size is relatively large.

Nevertheless, the limitations should be underlined: (1) The biggest limitation of this study is its retrospective nature. However, our inclusion and exclusion are rigorous. We perform a robust statistical analysis to control other confounding factors. Larger sample size is still needed to confirm our results and increase the statistical power. (2) The effect of the male partner’s MTHFR C677T polymorphism was not considered; (3) The combined effect of other genetic polymorphisms in the pathway of folic acid metabolism was not considered. (4) Serum folate and homocysteine levels were not detected, and their associations with IVF/ICSI outcomes could not be analyzed. Further studies are needed to evaluate the role of the male partner’s MTHFR genotype, the combined role of different genetic polymorphisms in the pathway of folic acid metabolism, and the interaction of serum vitamin B12, folate, hcy with MTHFR genotypes on ART outcomes. (5) Embryo factor at the first ET cycle was controlled. However, further study should compare the outcomes without controlling the embryo factor. (6) In consideration of the effect of genetic background. Only Chinese Han nationality was included in the study. Whether the conclusion could be extrapolated to other nationalities is not clear. (7) All the included women were regularly supplemented with 800 μg folic acid for three months. It is not clear whether supplementation of folic acid affects reproductive outcomes. The effect of folic acid supplementation on ART outcomes in patients with MTHFR 677T variant needs to be elucidated in the future. (8) The effect of MTHFR C677T polymorphism on reproductive outcomes under antagonist protocol or other protocols are not able to be analyzed in this study.

In summary, we think it is possibly worth examining MTHFR genotypes for the precision and safety on individualized COS strategy. Considering the combined effect of genetic factor and COS protocol, a protocol with a better ovarian response and safety may be foreseeable.

## Conclusions

MTHFR 677TT genotype is associated with decreased number of transferable embryos, decreased number of good-quality embryos, and a lower cumulative live birth rate in the first complete cycle in patients undergoing GnRHa short protocol. We should be very cautious when choosing GnRHa short protocol for women with MTHFR 677TT genotype.

## Supplementary Information

**Additional file 1:** **Supplementary Figure1.** Power estimation curve of each outcomes.

**Additional file 2:** **Supplementary Table 1.** Results of Multivariate analysis showing the association of MTHFR C677T genotype with the number of transferable embryos considering the interactive effect or not.

**Additional file 3:** **Supplementary Table 2.** Results of Multivariate analysis showing the association of MTHFR C677T genotype with the number of good-quality embryos considering the interactive effect or not.

**Additional file 4:** **Supplementary Table 3.** Results of Multivariate analysis showing the association of MTHFR C677T genotype with cumulative live birth considering the interactive effect or not.

**Additional file 5:** **Supplementary Table.** 4 The original non-matched data.

## Data Availability

The non-matched original data was deposited as a supplementary material in Supplementary Table 4. The original data and statistical R scripts and the statistical report are uploaded in our Github (https://github.com/minizenghong/MTHFRgenotype_and_CLBR).

## Abbreviations

MTHFR: 5,10-Methylenetetrahydrofolate reductase
IVF: In-vitro fertilization
ICSI: Intracytoplasmic sperm injection
ET: Embryo transfer
ART: Assisted reproduction technology
LBR: Live birth rate
CLBR: Cumulative live birth rate
