# Supplementary figures and images for: MTHFR 677TT is associated with decreased number of embryos and cumulative live birth rate in patients undergoing GnRHa short protocol: a retrospective study
Source: BMC Pregnancy Childbirth. 2022 Mar 1;22:170. doi: 10.1186/s12884-022-04506-4 (PMC8887009; doi:10.1186/s12884-022-04506-4)

## Statistical Power Estimation Curve for Each Outcome

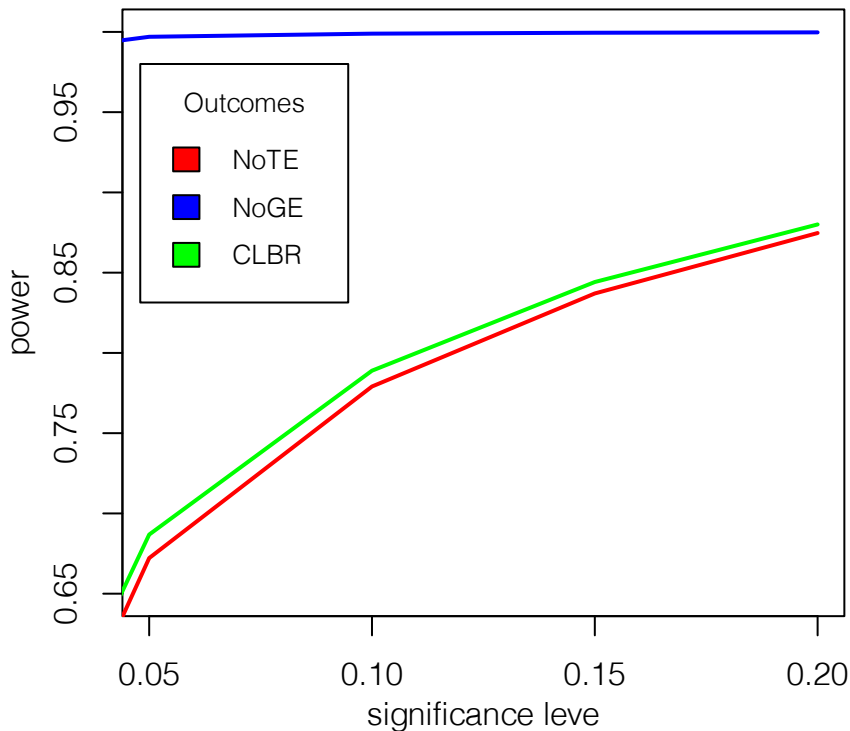

Supplement: Supplementary file 1 — Additional file 1: Supplementary Figure1. Power estimation curve of each outcomes. [file 12884_2022_4506_MOESM1_ESM.pdf]
