# Supplementary material for: MTHFR 677TT is associated with decreased number of embryos and cumulative live birth rate in patients undergoing GnRHa short protocol: a retrospective study
Source: BMC Pregnancy Childbirth. 2022 Mar 1;22:170. doi: 10.1186/s12884-022-04506-4 (PMC8887009; doi:10.1186/s12884-022-04506-4)
Supplement: Supplementary file 2 — Additional file 2: Supplementary Table 1. Results of Multivariate analysis showing the association of MTHFR C677T genotype with the number of transferable embryos considering the interactive effect or not. [file 12884_2022_4506_MOESM2_ESM.docx]

Supplementary Table 1 Results of Multivariate analysis showing the association of MTHFR C677T genotype with the number of transferable embryos considering the interactive effect or not.

|  | Not adjusted with interactive effect | | | Adjusted with interactive effect | | |
| --- | --- | --- | --- | --- | --- | --- |
|  | OR | 95%CI | P-value | OR | 95%CI | P-value |
| MTHFR C677T genotype |  |  |  |  |  |  |
| CC | Ref |  |  | Ref |  |  |
| CT | 0.97 | 0.91-1.03 | 0.282 | 0.96 | 0.89-1.03 | 0.278 |
| TT | 0.91 | 0.82-1.00 | 0.055 | 0.99 | 0.88-1.11 | 0.840 |
| Age |  |  |  |  |  |  |
| <35 | Ref |  |  | Ref |  |  |
| >=35 | 0.95 | 0.88-1.02 | 0.141 | 0.95 | 0.88-1.02 | 0.133 |
| BMI |  |  |  |  |  |  |
| 18.5-20 | Ref |  |  | Ref |  |  |
| 20-23 | 0.94 | 0.88-1.01 | 0.081 | 0.95 | 0.88-1.01 | 0.101 |
| 23-25 | 0.87 | 0.81-0.95 | 0.001 | 0.88 | 0.81-0.95 | 0.001 |
| Infertility cause |  |  |  |  |  |  |
| Male factor | Ref |  |  | Ref |  |  |
| Female factor | 1.11 | 1.00-1.22 | 0.052 | 1.10 | 1.00-1.22 | 0.060 |
| Infertility type |  |  |  |  |  |  |
| Primary infertility | Ref |  |  | Ref |  |  |
| Secondary infertility | 1.07 | 1.01-1.14 | 0.021 | 1.07 | 1.01-1.14 | 0.023 |
| Protocol |  |  |  |  |  |  |
| Long protocol | Ref |  |  | Ref |  |  |
| Short protocol | 1.03 | 0.97-1.09 | 0.403 | 1.04 | 0.95-1.13 | 0.407 |
| Interactive effect |  |  |  |  |  |  |
| CT:Short protocol |  |  |  | 1.03 | 0.91-1.16 | 0.649 |
| TT:Short protocol |  |  |  | 0.76 | 0.61-0.94 | 0.013 |

Note: Poisson regression model was used to calculate the OR and 95%CI. MTHFR = 5,10-methylenetetrahydrofolate reductase, CI = confidence interval, OR = odds ratio, Ref = reference. The model was adjusted for age, BMI, stimulation protocol, infertility cause, and infertility type. P for interaction: 0.020.
