# Supplementary material for: MTHFR 677TT is associated with decreased number of embryos and cumulative live birth rate in patients undergoing GnRHa short protocol: a retrospective study
Source: BMC Pregnancy Childbirth. 2022 Mar 1;22:170. doi: 10.1186/s12884-022-04506-4 (PMC8887009; doi:10.1186/s12884-022-04506-4)
Supplement: Supplementary file 3 — Additional file 3: Supplementary Table 2. Results of Multivariate analysis showing the association of MTHFR C677T genotype with the number of good-quality embryos considering the interactive effect or not. [file 12884_2022_4506_MOESM3_ESM.docx]

Supplementary Table 2 Results of Multivariate analysis showing the association of MTHFR C677T genotype with the number of good-quality embryos considering the interactive effect or not.

|  | Not adjusted with interactive effect | | | Adjusted with interactive effect | | |
| --- | --- | --- | --- | --- | --- | --- |
|  | OR | 95%CI | P-value | OR | 95%CI | P-value |
| MTHFR C677T genotype |  |  |  |  |  |  |
| CC |  |  |  |  |  |  |
| CT | 0.97 | 0.91-1.03 | 0.357 | 0.96 | 0.89-1.04 | 0.316 |
| TT | 0.88 | 0.79-0.97 | 0.015 | 0.97 | 0.86-1.10 | 0.668 |
| Age |  |  |  |  |  |  |
| <35 |  |  |  |  |  |  |
| >=35 | 0.94 | 0.87-1.01 | 0.104 | 0.94 | 0.87-1.01 | 0.097 |
| BMI |  |  |  |  |  |  |
| 18.5-20 |  |  |  |  |  |  |
| 20-23 | 0.94 | 0.88-1.01 | 0.111 | 0.95 | 0.88-1.02 | 0.141 |
| 23-25 | 0.86 | 0.79-0.94 | 0.001 | 0.86 | 0.79-0.94 | 0.001 |
| Infertility cause |  |  |  |  |  |  |
| Male factor |  |  |  |  |  |  |
| Female factor | 1.13 | 1.02-1.26 | 0.025 | 1.12 | 1.01-1.25 | 0.031 |
| Infertility type |  |  |  |  |  |  |
| Primary infertility |  |  |  |  |  |  |
| Secondary infertility | 1.01 | 0.95-1.08 | 0.686 | 1.03 | 0.94-1.13 | 0.568 |
| Protocol |  |  |  |  |  |  |
| Long protocol |  |  |  |  |  |  |
| Short protocol | 1.06 | 0.99-1.13 | 0.073 | 1.06 | 0.99-1.13 | 0.080 |
| Interactive effect |  |  |  |  |  |  |
| CT:Short protocol |  |  |  | 1.03 | 0.91-1.17 | 0.600 |
| TT:Short protocol |  |  |  | 0.71 | 0.56-0.89 | 0.004 |

Note: Poisson regression model was used to calculate the OR and 95%CI. MTHFR = 5,10-methylenetetrahydrofolate reductase, CI = confidence interval, OR = odds ratio, Ref = reference. The model was adjusted for age, BMI, stimulation protocol, infertility cause, and infertility type. P for interaction: 0.005.
