# Supplementary material for: MTHFR 677TT is associated with decreased number of embryos and cumulative live birth rate in patients undergoing GnRHa short protocol: a retrospective study
Source: BMC Pregnancy Childbirth. 2022 Mar 1;22:170. doi: 10.1186/s12884-022-04506-4 (PMC8887009; doi:10.1186/s12884-022-04506-4)
Supplement: Supplementary file 4 — Additional file 4: Supplementary Table 3. Results of Multivariate analysis showing the association of MTHFR C677T genotype with cumulative live birth considering the interactive effect or not. [file 12884_2022_4506_MOESM4_ESM.docx]

Supplementary Table 3 Results of Multivariate analysis showing the association of MTHFR C677T genotype with cumulative live birth considering the interactive effect or not.

|  | Not adjusted with interactive effect | | | Adjusted with interactive effect | | |
| --- | --- | --- | --- | --- | --- | --- |
|  | HR | 95%CI | P-value | HR | 95%CI | P-value |
| MTHFR genotype |  |  |  |  |  |  |
| CC | 1.00 | ref |  |  |  |  |
| CT | 1.19 | 1.19(1.04-1.37) | 0.012 | 1.14 | 0.93-1.40 | 0.194 |
| TT | 1.23 | 1.23(0.99-1.53) | 0.060 | 0.94 | 0.69-1.28 | 0.693 |
| Age |  |  |  |  |  |  |
| Age<35 | 1.00 | ref |  |  |  |  |
| Age≥35 | 1.17 | 1.17(1.01-1.36) | 0.035 | 1.18 | 1.01-1.36 | 0.032 |
| BMI |  |  |  |  |  |  |
| 18.5-20 | 1.00 | ref |  |  |  |  |
| 20-23 | 0.96 | 0.96(0.83-1.12) | 0.592 | 0.95 | 0.82-1.10 | 0.494 |
| 23-25 | 1.01 | 1.01(0.85-1.21) | 0.881 | 1.00 | 0.84-1.20 | 0.974 |
| Infertility cause |  |  |  |  |  |  |
| Male factor | 1.00 | ref |  |  |  |  |
| Female factor | 1.02 | 1.02(0.79-1.33) | 0.858 | 1.03 | 0.80-1.33 | 0.828 |
| Protocol |  |  |  |  |  |  |
| Long protocol | 1.00 | ref |  |  |  |  |
| Short protocol | 1.21 | 1.21(1.06-1.38) | 0.006 | 1.08 | 0.87-1.34 | 0.485 |
| Infertility type |  |  |  |  |  |  |
| Primary infertility | 1.00 | ref |  |  |  |  |
| Secondary infertility | 1.07 | 1.07(0.93-1.23) | 0.371 | 1.06 | 0.92-1.22 | 0.419 |
| Interactive effect |  |  |  |  |  |  |
| CT:Short protocol |  |  |  | 1.09 | 0.83-1.44 | 0.546 |
| TT:Short protocol |  |  |  | 1.92 | 1.28-2.89 | 0.002 |

Note: Cox regression model was used to calculate the HR and 95%CI. MTHFR = 5,10-methylenetetrahydrofolate reductase, CI = confidence interval, OR = odds ratio, Ref = reference. The model was adjusted for age, BMI, stimulation protocol, infertility cause, and infertility type. P for interaction: 0.013.
